# Carrier control in 2D transition metal dichalcogenides with Al_2_O_3_ dielectric

**DOI:** 10.1038/s41598-019-45392-9

**Published:** 2019-06-19

**Authors:** Chit Siong Lau, Jing Yee Chee, Dickson Thian, Hiroyo Kawai, Jie Deng, Swee Liang Wong, Zi En Ooi, Yee-Fun Lim, Kuan Eng Johnson Goh

**Affiliations:** 1 Institute of Materials Research and Engineering (IMRE), Agency for Science, Technology and Research (A*STAR), 2 Fusionopolis Way, Singapore, 138634 Singapore; 2 Institute of High Performance Computing (IHPC), Agency for Science, Technology and Research (A*STAR), 1 Fusionopolis Way, Singapore, 138632 Singapore; 3 Department of Physics, National University of Singapore, 2 Science Drive 3, Singapore, 117551 Singapore

**Keywords:** Two-dimensional materials, Two-dimensional materials

## Abstract

We report transport measurements of dual gated MoS_2_ and WSe_2_ devices using atomic layer deposition grown Al_2_O_3_ as gate dielectrics. We are able to achieve current pinch-off using independent split gates and observe current steps suggesting possible carrier confinement. We also investigated the impact of gate geometry and used electrostatic potential simulations to explain the observed device physics.

## Introduction

Two-dimensional transition metal dichalcogenides (2D TMDCs) have attracted increasing interest for creating novel quantum electronics due to their intriguing properties^1–3^. Notably, these atomically thin materials provide natural charge confinement in one spatial dimension and can also possess a sizable bandgap, offering unique opportunities for fabricating novel electrostatically gated devices for quantum information processing^4,5^. The smooth confinement potential minimizes edge and defect states that can otherwise arise from using physical etching processes. Recently, theoretical works have explored exploiting spin and valley states in 2D TMDCs to create electrostatically gated spin-valley qubits^6–8^. This is promising due to the broken spatial inversion symmetry in 2D TMDCs and strong spin-valley coupling that can allow for concurrently valley and spin-polarized charge carriers with long coherence times. Furthermore, valley-dependent optical selection rule in 2D TMDCs suggests the possibility of creating optically controllable spin-valley qubits in 2D TMDCs^8–10^.

The first step towards such TMDC quantum devices is to demonstrate effective carrier confinement with electrostatically tunable confinement potentials. However, it is technically challenging to implement effective carrier confinement in 2D TMDCs due to issues such as residual disorder, low mobility and non-ohmic contacts to the TMDC^11^. While van-der-Waals heterostructure devices utilizing encapsulated hexagonal boron nitride (hBN) have demonstrated effective carrier confinement with electrostatic gates and superior performance, scalability can be limited. Carrier confinement has been demonstrated in MoS_2_ devices using hBN at temperatures up to 4 K^12–16^. WSe_2_ devices using ALD oxides have demonstrated carrier confinement only at 240 mK^17^. Measurements at such millikelvin temperatures can be a costly experimental challenge. Here, we present our work on 2D MoS_2_ and WSe_2_ devices encapsulated with atomic layer deposition (ALD) Al_2_O_3_ using a 4 K cryostat. We observe current pinch-off and current steps up to 25 K suggesting carrier confinement while demonstrating independent split top gate control over the current flow. Using electrostatic potential simulations, our device physics can be consistently explained by the electrostatic potential variations resulting from the combination of our device geometries and applied gate voltages.

## Experiment

**Figure 1:**
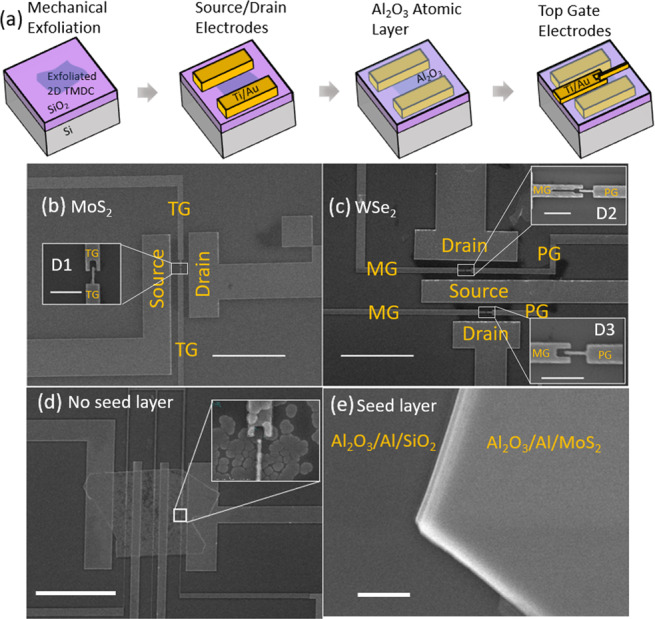
Device fabrication. (**a**) Schematic of the device fabrication process. SEM images of the few-layer (**b**) MoS_2_ and the (**c**) WSe_2_ devices. The insets show closeups of the split gate geometries. They are labelled TG, MG and PG. Scale bars are 10 μm and 500 nm (insets). SEM images of the ALD growth (**d**) without and (**e**) with an Al seed layer. Scale bars are (**d**) 10 μm (**e**) and 500 nm.

The device fabrication process is illustrated in Fig. 1a. SEM images of the fabricated devices are shown in Fig. 1b (MoS_2_) and 1c (WSe_2_). We fabricated our devices by mechanically exfoliating flakes from bulk crystals. These flakes are deposited on to heavily doped silicon substrates with a 285 nm layer of SiO_2_ on top. The substrates also function as global back gates for tuning the overall carrier densities in the conducting materials. We next optically identified appropriate few-layer flakes of suitable size for device fabrication. Using standard e-beam lithography and e-beam evaporation, we deposited 10/80 nm of Ti/Au metal as source-drain electrodes. We maximized the contact areas of the electrodes in order to achieve good ohmic contact. After lift-off, the samples are annealed at 200 °C in forming gas H_2_/Ar atmosphere for 2 hours to improve the contacts and remove residual resist^18^.

To form local top gates essential for inducing carrier confinement, a suitable dielectric layer is required. We used a Beneq TFS200 ALD system with precurors Trimehylaluminium (TMA) and water at a temperature of 200 °C to deposit 33 nm of Al_2_O_3_ high-*k* dielectric. While 2D TMDCs offer a pristine surface and lack of dangling bonds, this negatively affects the ALD process as it results in island-type growth which leads to poor dielectric coverage and gate leakage (Fig. 1d). To circumvent this problem, we first deposited a 1 nm thick Al seed layer via thermal evaporation prior to ALD growth which results in uniform ALD coverage^19^. The two top split gates were subsequently defined using e-beam lithography and e-beam evaporation to deposit 5/30 nm of Ti/Au metal. After lift-off, the devices were annealed once again at 200 °C in forming gas H_2_/Ar atmosphere for 2 hours.

## Results and Discussion

We measured a total of three devices labelled D1, D2 and D3. As the WSe_2_ flake obtained is sufficiently large, we patterned two devices with different geometries on the same flake (D2, D3) (Fig. 1c inset). D2 was patterned with a gap in the center of the gate labelled MG. Previous works on 2DEG quantum dots have raised the necessity of having independent gates to control multiple tunneling barriers, resulting in gaps between the confinement gates^12,14^. It remains unclear how gaps in device geometries can impact the transport properties and confinement potential of the device. We investigated the influence of this gap by comparing the characteristics of D2 and D3. While the lithographically defined gate geometries suggest that tunnelling barriers will likely be formed in the two gaps between the gates PG and MG (insets of Fig. 1b,c), our simulations, discussed later, show that the actual electrostatic potential profile can be different from the gate geometries.

**Figure 2:**
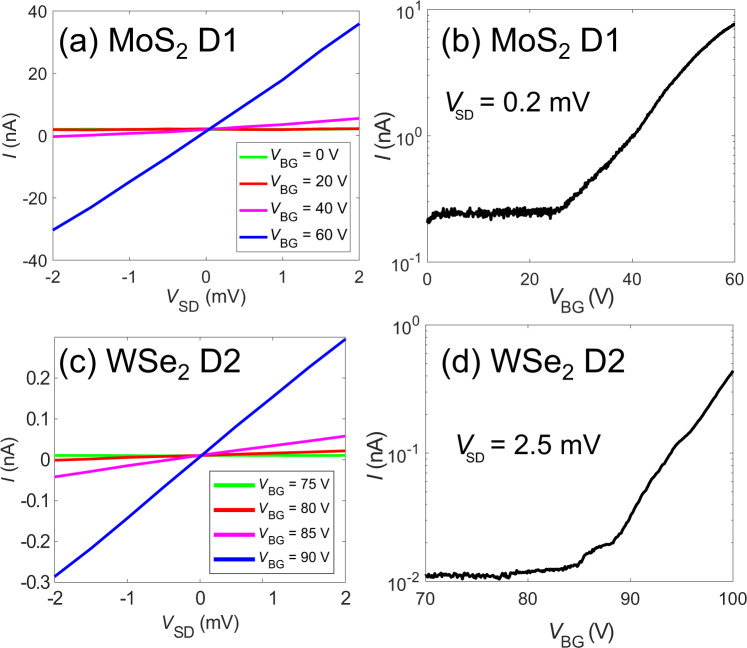
Transport properties. Current *I* vs source-drain voltage *V*_SD_ curves at various applied back gate voltages *V*_BG_ for (**a**) MoS_2_ (D1) and (**c**) WSe_2_ (D2). Current *I* vs back-gate voltage *V*_BG_ curves for (**b**) MoS_2_ (D1) and (**d**) WSe_2_ (D2). The current starts to increase at 23 V for MoS_2_ D1 and 84 V WSe_2_ D2 as the devices are turned on.

We first measured transport without using the top local split gates. All measurements were performed in a closed-cycle Janis cryostat using low-noise Stanford Research Systems SRS928 voltage sources and an AlazarTech ATS9440 DAQ at 4 K, unless otherwise stated. After cooling to 4 K, the devices were illuminated using a 660 nm light-emitting diode to reduce defect-related impurity traps and improve the carrier homogeneity. All subsequent measurements are performed with the light emitting diode turned off^14,20^. Figure 2 shows the transport properties of devices D1 and D2. For both MoS_2_ D1 and WSe_2_ D2, we recorded linear current *I* versus source-drain voltage *V*_SD_ curves at various back gate voltages *V*_BG_ indicating ohmic contacts that are important so that the contacts do not dominate device operation (Fig. 2a,c). Characteristic n-type semiconducting behaviors are observed for both devices (Fig. 2b,d), but with different turn on voltages. WSe_2_ D2 and D3 (Supplementary Material) have more positive turn on voltages compared to MoS_2_ D1, which leads to distinct operating top gate voltages for the two materials, as will be shown in the subsequent sections.

**Figure 3:**
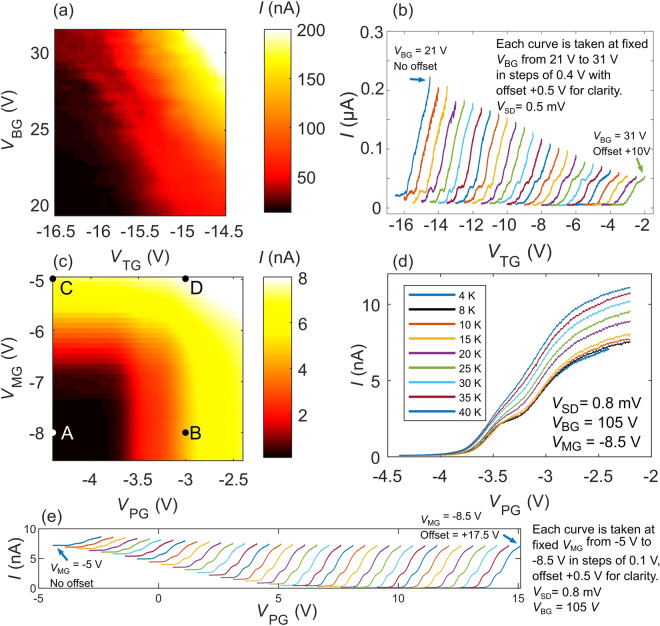
Top gate control. (**a**) Current *I* vs top gate voltage *V*_TG_ and back gate voltage *V*_BG_ for MoS_2_ D1. The current can be smoothly tuned by both *V*_TG_ and *V*_BG_. The dark lower left region highlights the voltage space when the conducting channel of the device is pinched off. (**b**) *I* vs *V*_TG_ at various applied *V*_BG_, where current steps can be observed, suggesting the formation of a quantum constriction. (**c**) Current *I* vs top gate voltages *V*_MG_ and *V*_PG_ at *V*_BG_ = 105 V for WSe_2_ D2 (see Fig. 1c inset). The current through the device can be independently controlled by the split top gates PG and MG. (**d**) Current *I* vs *V*_PG_ at fixed *V*_BG_ and *V*_MG_ taken at different temperatures. The current steps are visible up to 25 K. (**e**) *I* vs *V*_PG_ at various applied *V*_MG_ and fixed *V*_BG_ = 105 V, where similar current steps are observed.

To achieve carrier confinement, the carrier densities in our TMDCs should be locally tunable with the top gates. Figure 3a shows the current *I* through MoS_2_ D2 as a function of both the applied back gate voltage *V*_BG_ and top gate voltage *V*_TG_. In this measurement, the same voltage *V*_TG_ was applied to both the split gates (inset of Fig. 1b). The current through the device depends on both *V*_BG_ and *V*_TG_ and can be pinched off when sufficiently negative *V*_TG_ was applied (lower left dark region). The required *V*_TG_ to achieve pinch-off becomes increasingly negative at more positive *V*_BG_, i.e. at higher carrier densities.

In dual gated systems, signatures of quantum confinement reported in literature are the following. (1) Regular and periodically spaced Coulomb diamonds/conductance peaks which occur in quantum dots with two tunnelling barriers^12,14,17,21^. (2) Multiple regularly spaced step like features in multiples of *e*^2^/*h* that occur in quantum constrictions with symmetric top gates reflecting conductance quantisation^13,22^. (3) Few/single current steps in device geometries with asymmetric top gates that occurs at similar conductance for several back-gate voltages^14,15^.

From the *I* vs *V*_TG_ curves for fixed *V*_BG_ (Fig. 3b), we observe current steps of similar conductances at multiple *V*_BG_ suggesting possible carrier confinement as the width of the conducting channel is reduced with increasing *V*_TG_, similar to measurements reported in other works^13–15^. Small current peaks are also observed. Such peaks have been observed in other material systems and can have various origins such as universal conductance fluctuations, Fabry perot oscillations and single-electron tunnelling^12,14,17,21,23–28^. Carrier disorder in our devices can also possibly lead to the observed peaks^14^. Figure 3 presents two-terminal measurements and can include significant contact resistance. Subtracting these parasitic resistances is difficult as they can be strongly gate and bias voltage dependent. Similar issues have been observed in refs ^14,15,22^, where the authors likewise observe steps at conductances smaller than the quantum conductance. We note that in order to gain deeper insight and demonstrate exact quantization, further improvements in the device and material quality (carrier homogeneity, impurities, dielectric interfaces and contact quality) as well as the optimization of device geometry and experimental setup (e.g. 4-probe, lower temperatures) will be required and represent on going efforts in the group.

We next performed similar measurements on our WSe_2_ devices D2 (Fig. 3c,d) and D3 (Supplementary Material). To confirm that the current through the device is due to electrostatic modification of the carrier density by the top gates, we measured the device current while applying independent voltages *V*_MG_ and *V*_PG_ to the top gate electrodes MG and PG (inset Fig. 1c). Figure 3c shows *I* as a function of *V*_MG_ and *V*_PG_ at a fixed *V*_BG_ = 105 V. We observe that the current is pinched off only when *both V*_MG_ and *V*_PG_ are sufficiently negative, leading to the rectangular dark pinch-off region in the lower left of Fig. 3c. The edges of this rectangular pinch-off region are non-diagonal, indicating that current can be independently tuned with minimal cross-capacitance. We note that the top gate voltages applied in WSe_2_ devices D2 and D3 (~−8.5 V) are smaller than the devices applied in MoS_2_ D1 (~−16.5 V). This difference in applied top gate voltages is due to the different turn on voltages for MoS_2_ and WSe_2_ (Fig. 2b,d). The larger *V*_BG_ required to turn on WSe_2_ compared to MoS_2_ consequently means that lower voltages are required to deplete the carrier distribution under the top gate electrodes. Thus, even though similar device architectures can be applied across different 2D TMDC materials to achieve carrier confinement, design considerations should take into account intrinsic material properties such as material doping levels that can depend on the dielectric type.

When varying a single gate voltage *V*_PG_, similar current steps observed in MoS_2_ D1 are likewise discerned in WSe_2_ D2 (Fig. 3e). At larger *V*_MG_ (*V*_MG_ = −6.5 V to −8.5 V), the steps occur at similar conductances, suggesting the formation of a carrier constriction. At smaller *V*_MG_ (*V*_MG_ = −5 V to −6.5 V), the conductance of the current steps deviates, likely due to the constriction being ill defined as a result of the smaller applied *V*_MG_. Figure 3d shows the temperature dependence of the *I* versus *V*_PG_ curves taken at different temperatures from 4 K to 40 K. The current steps gradually smear out with increasing temperature, consistent with thermal broadening of the electron energy distribution.

Similar pinch-off characteristics are observed in device D3 but with two main differences. Firstly, to achieve current pinch-off, a more negative *V*_PG_ ≈ −6.5 V was required for D3 compared to D2 (*V*_PG_ ≈ −3.5 V, *V*_MG_ for both D2 and D3 are similar at ≈−6.5 V). Secondly, there are no obvious current steps in D3. These differences can be due factors including inhomogeneity of the carrier distribution or the electrostatic potential landscape. A more interesting possibility is the difference in the gate geometries between D2 and D3; D2 is fabricated with a gap in the middle of the gate MG. We further investigated this possibility through simulations of the electrostatic potentials. While we can control current flow using our split top gates MG and PG and observe possible carrier confinement in the TMDC, the exact geometries of any quantum constrictions are difficult to extract from the transport measurements. To gain a deeper insight into our device physics and the geometries of possible quantum constrictions, we used the commercial software COMSOL to perform a finite element analysis simulation of the electrostatic potential profiles.

**Figure 4:**
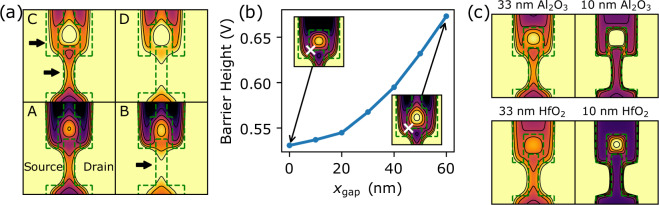
Electrostatic potential simulations. Top gate geometries are overlaid as dotted green lines. (**a**) Electric potential simulations *U*(*x*, *y*) of WSe_2_ device D2, The color scale is in arbitrary units as the potential is generally tunable with the back-gate. Darker colors correspond to more positive *U* and lower electron density. Panels (A–D) show *U*(*x*, *y*) with top gate voltages corresponding to the labels in Fig. 3c. A - device pinched off, B - conductance through one quantum constriction, (**C**) conductance through two possible quantum constrictions. D - field effect regime. (**b**) Potential barrier height vs *x*_gap_, size of the gap in the gate MG. Insets - closeups of *U* with the position of barrier height indicated (Supplementary Fig. 2). (**c**) Dependence of *U*(*x*, *y*) on the ALD dielectric layer. All top gate voltages are set to 0 V.

We begin by investigating how the electrostatic potential in WSe_2_ D2 is dependent on gate electrodes MG and PG. Figure 4a shows the electrostatic potential profiles for WSe_2_ D2 at different *V*_MG_ and *V*_PG_ corresponding to the positional labels marked A–D in Fig. 3c. We note that the actual device potentials are not easily calculated due to difficulty in determining the actual WSe_2_ intrinsic doping. Nevertheless, the simulations suggest how the electron density profiles depend on the gates. At position A, the electrostatic potential profile shows no available conduction channel resulting in complete current pinch-off. When *V*_PG_ is made less negative relative to *V*_MG_, a conduction channel (arrow in panel B) is opened. The formation of this quantum constriction is marked by the conduction step in the current (Fig. 3d). At position C, when V_MG_ is less negative relative to position A for the same V_PG_, the barrier potential decreases slightly and a possible parallel conductance channel (arrows in Fig. 4a, panel C) can open, leading to increased current. Finally, when both *V*_PG_ and *V*_MG_ are made less negative at position D compared to A, current flow is no longer limited by the constrictions and the device operates in a field effect regime.

We next investigated the difference in transport characteristics of WSe_2_ D2 and D3. Our simulations show that the presence of the gap in gate MG of device D2 may lead to stronger carrier confinement. Figure 4b shows the potential barrier of the constriction (arrows) as a function of the MG gap size *x*_gap_ for the same applied gate voltages. This is a possible reason why no obvious current steps are measured in D3 (*x*_gap_ = 0 nm), and a more negative *V*_PG_ compared to D2 is required to pinch off the current.

Finally, the potential can be more sharply defined by reducing the thickness of the ALD Al_2_O_3_ to obtain quantum dots (Fig. 4c). This can also be achieved with a higher-*k* dielectric such as HfO_2_. At the same thickness of 33 nm, a HfO_2_ dielectric produces an electrostatic potential profile with only a single quantum constriction. Such flexibility is a possible advantage of ALD dielectrics. TMDC based quantum devices can be designed with desired properties by precise control of the ALD dielectric type and thickness.

## Conclusion

Electrostatic control over carrier confinement is a crucial step towards realizing novel TMDC-based quantum electronics such as spin-valley qubits. We present measurements and simulations of 2D MoS_2_ and WSe_2_ devices with local split top gates. Our devices are made using ALD grown Al_2_O_3_. We can achieve current pinch-off and observe current steps and peaks suggesting possible carrier confinement at 4 K, similar to measurements at lower temperatures of more complex van-der-Waals heterostructure TMDC devices based on exfoliated hexagonal boron nitride. Our device physics and transport characteristics can be explained through simulations of the electrostatic potential variations resulting from the combination of the device geometries and applied gate voltages. Future work can explore the possibility of using large-area TMDC sheets grown with chemical vapour deposition. With further improvements in ALD growth and contact quality, scalable TMDC quantum devices fabricated using CMOS-compatible technology remain a distinct possibility.

## Supplementary information

Supplementary Information
