# Author Correction: Carrier control in 2D transition metal dichalcogenides with Al_2_O_3_ dielectric

**DOI:** 10.1038/s41598-021-96557-4

**Published:** 2021-08-25

**Authors:** Chit Siong Lau, Jing Yee Chee, Dickson Thian, Hiroyo Kawai, Jie Deng, Swee Liang Wong, Zi En Ooi, Yee-Fun Lim, Kuan Eng Johnson Goh

**Affiliations:** 1 Institute of Materials Research and Engineering (IMRE), Agency for Science, Technology and Research (A*STAR), 2 Fusionopolis Way, Singapore, 138634 Singapore; 2 Institute of High Performance Computing (IHPC), Agency for Science, Technology and Research (A*STAR), 1 Fusionopolis Way, Singapore, 138632 Singapore; 3 Department of Physics, National University of Singapore, 2 Science Drive 3, Singapore, 117551 Singapore

Correction to: *Scientific Reports * 10.1038/s41598-019-45392-9, published online 19 June 2019

The original version of this Article contained an error in Figure 3b, where the y-axis

“/(nA)”

now reads:

“/(μA)”

The original Figure 3 and accompanying legend appear below. The original Article has been corrected.

**Figure 3:**
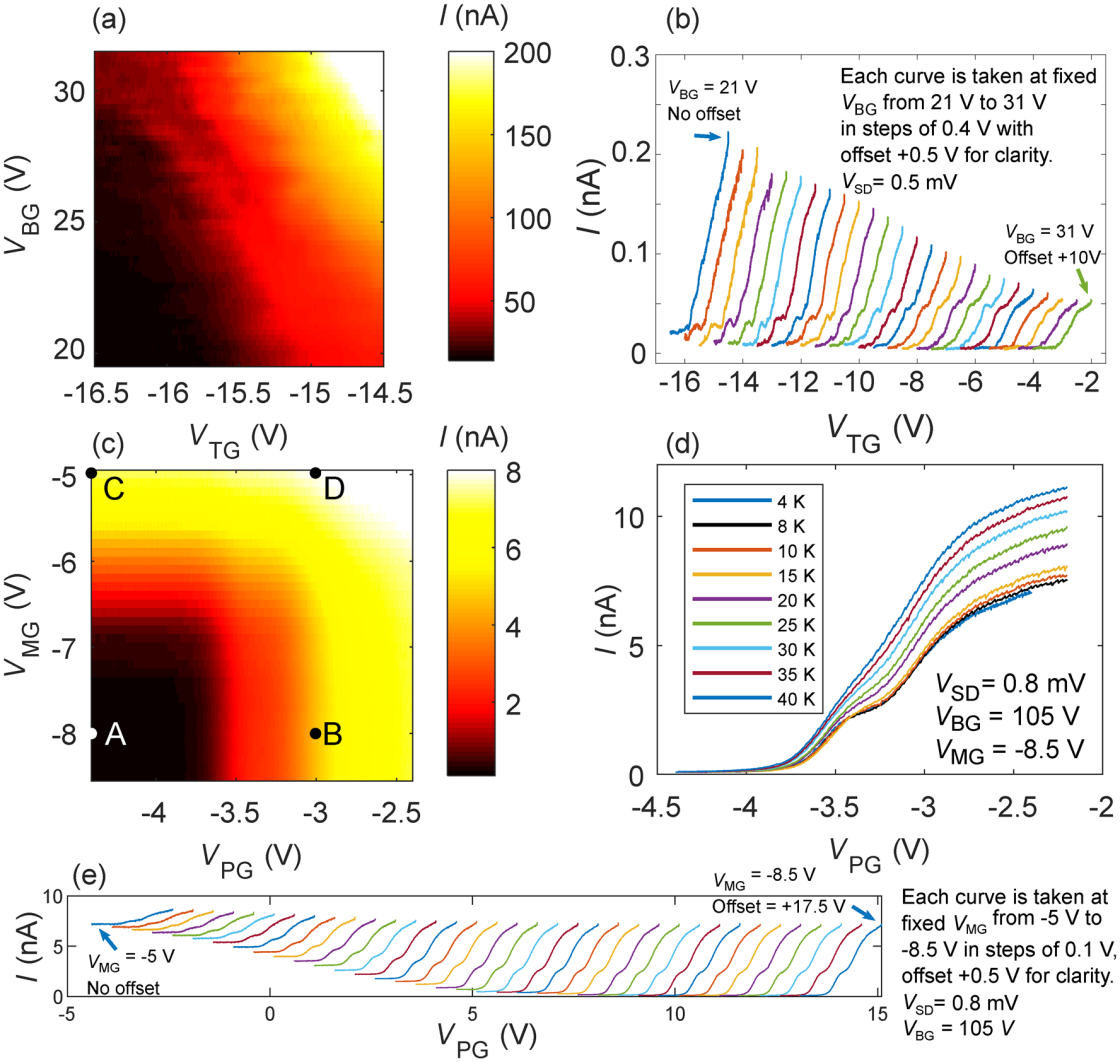
Top gate control. (**a**) Current *I* vs top gate voltage *V*_TG_ and back gate voltage *V*_BG_ for MoS_2_ D1. The current can be smoothly tuned by both *V*_TG_ and *V*_BG_. The dark lower left region highlights the voltage space when the conducting channel of the device is pinched off. (**b**) *I* vs *V*_TG_ at various applied *V*_BG_, where current steps can be observed, suggesting the formation of a quantum constriction. (**c**) Current *I* vs top gate voltages *V*_MG_ and *V*_PG_ at *V*_BG_ = 105 V for WSe_2_ D2 (see Fig. 1c inset). The current through the device can be independently controlled by the split top gates PG and MG. (**d**) Current *I* vs *V*_PG_ at fixed *V*_BG_ and *V*_MG_ taken at different temperatures. The current steps are visible up to 25 K. (**e**) *I* vs *V*_PG_ at various applied *V*_MG_ and fixed *V*_BG_ = 105 V, where similar current steps are observed.

