# Supplementary material for: Carrier control in 2D transition metal dichalcogenides with Al2O3 dielectric
Source: Sci Rep. 2019 Jun 19;9:8769. doi: 10.1038/s41598-019-45392-9 (PMC6584693; doi:10.1038/s41598-019-45392-9)
Supplement: Supplementary file 1 — Supplementary Information [file 41598_2019_45392_MOESM1_ESM.pdf]

Supplementary Material

Chit Siong Lau,[1] Jing Yee Chee,[1] Dickson Thian,[1] Hiroyo Kawai,[2] Jie Deng,[1] Swee Liang Wong,[1] Zi En Ooi,[1] Yee-Fun Lim,[1] and Kuan Eng Goh Johnson[3,1,a]

[1] *Institute of Materials Research and Engineering (IMRE), Agency for Science, Technology and Research (A*STAR), 2 Fusionopolis Way, 138634, Singapore*

[2] *Institute of High Performance Computing (IHPC), Agency for Science, Technology and Research (A*STAR), 1 Fusionopolis Way, 138632, Singapore*

[3] *Department of Physics, National University of Singapore, 2 Science Drive 3, 117551, Singapore*

[a] kejgoh@yahoo.com

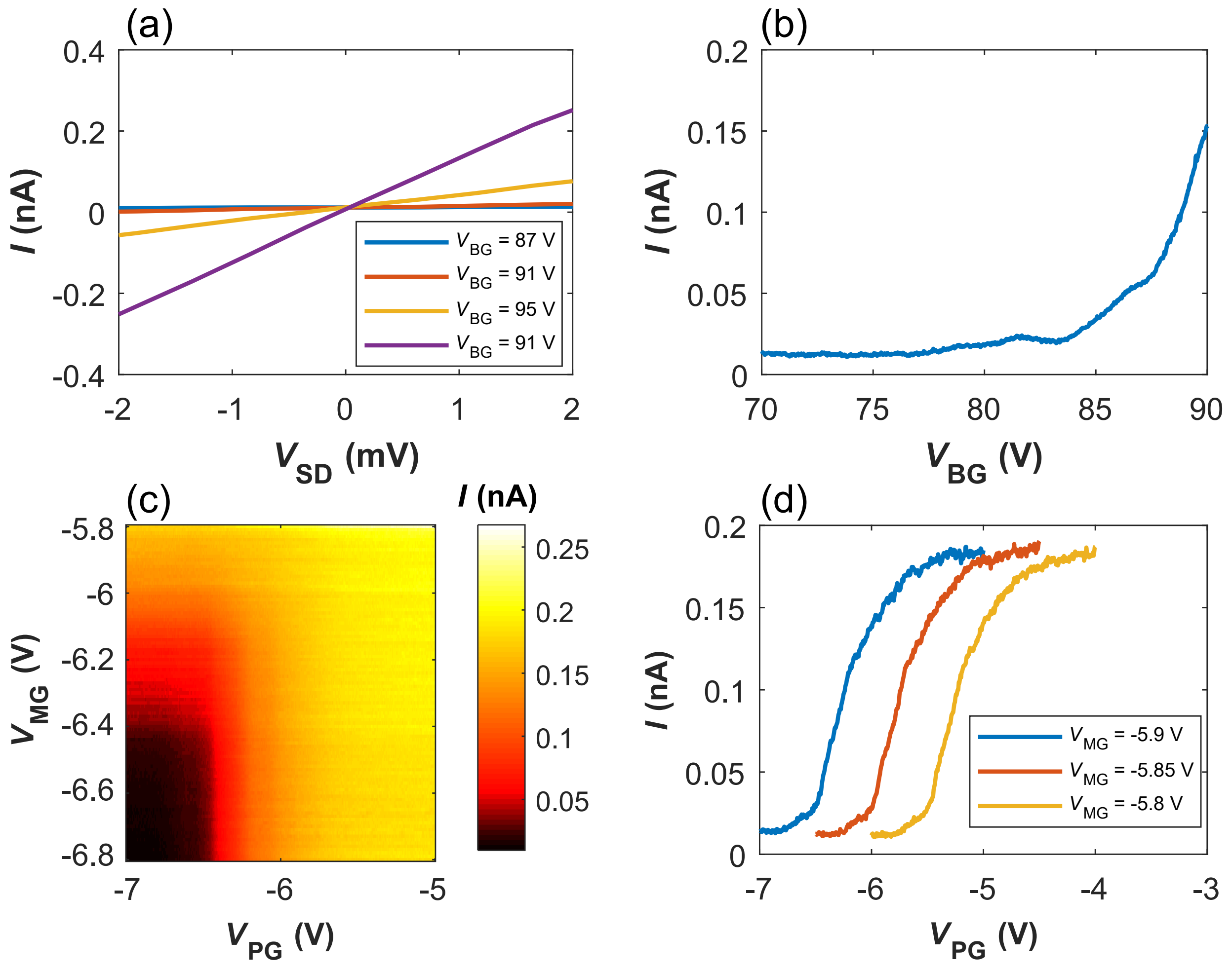

FIG. 1. **Transport measurements of WSe_2 D3.** (a) Current I vs source-drain voltage V_{SD} at various applied back gate voltages V_{BG}. (b) Current I vs back gate voltage V_{BG}. Typical n-type behaviour is observed. (c) Current I vs top gate voltages V_{MG} and V_{PG}. (d) Current I vs V_{PG} at fixed V_{BG} for different V_{MG}. The curves are offset for clarity.

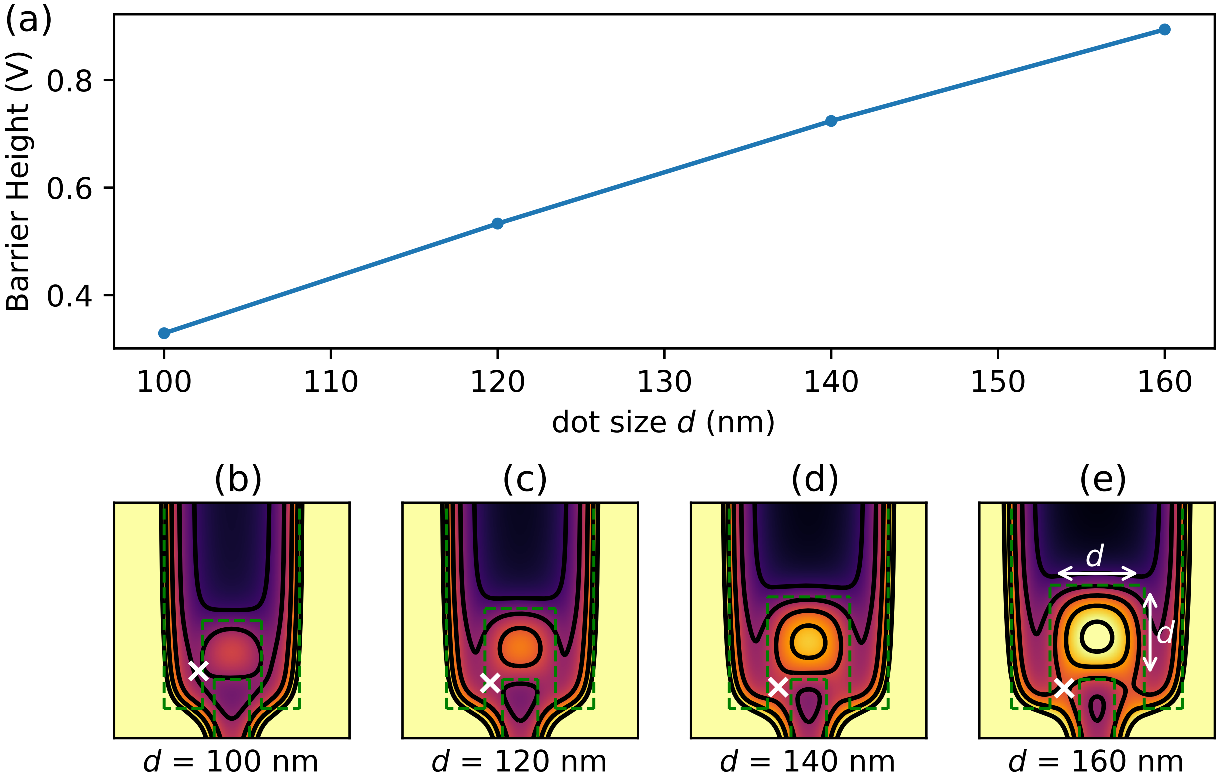

FIG. 2. **Quantum dot barrier heights** (a) Simulation results showing how the 2D quantum dot barrier height depends on the quantum dot size, as defined by the physical dimensions d. (b-e) 2D maps of electrostatic potential in arbitrary units, with darker colors showing regions of higher potential and lower electron density. The quantum dot barrier height is defined from the position of the potential saddle point (marked with an X) to the maximum potential of the dot.The top gate geometries are shown overlaid with green dotted lines.

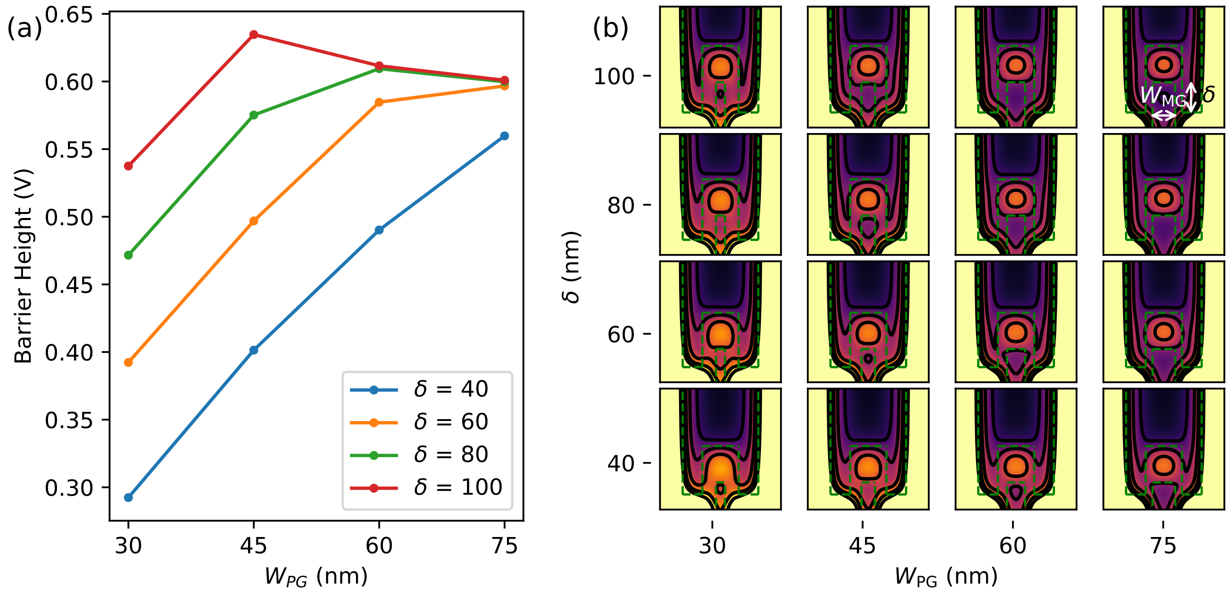

FIG. 3. **Geometric dependence of barrier heights** (a) Simulation results showing how the 2D quantum dot barrier height depends non-trivially on the geometry of the gates. For a given dot size, optimization of the dot geometry and structure is crucial to maximize quantum confinement (b) 2D maps of electrostatic potential overlaid on the simulated top gate geometry (dotted, green) in arbitrary units. The parameters δ and $W_{\rm PG}$ are defined in the top right plot of (b).
